# Optimising blood glucose control with portioned meal box in type 2 diabetes mellitus patients: a randomised control trial

**DOI:** 10.3389/fnut.2023.1216753

**Published:** 2023-07-18

**Authors:** Tanu-udom Maneesing, Atchara Dawangpa, Pechngam Chaivanit, Sudjai Songsakul, Piyapong Prasertsri, Natália Yumi Noronha, Lígia Moriguchi Watanabe, Carla Barbosa Nonino, Busadee Pratumvinit, Chanachai Sae-Lee

**Affiliations:** ^1^Faculty of Allied Health Sciences, Burapha University, Chonburi, Thailand; ^2^Exercise and Nutrition Innovation and Sciences Research Unit, Burapha University, Chonburi, Thailand; ^3^Department of Clinical Pathology, Faculty of Medicine Siriraj Hospital, Mahidol University, Bangkok, Thailand; ^4^Research Division, Faculty of Medicine Siriraj Hospital, Mahidol University, Bangkok, Thailand; ^5^Department of Internal Medicine, Faculty of Medicine, Burapha University, Chonburi, Thailand; ^6^Department of Health Sciences, Ribeirão Preto Medical School, São Paulo, Brazil; ^7^Department of Internal Medicine, Ribeirão Preto Medical School, University of São Paulo, São Paulo, Brazil

**Keywords:** portioned meal box, dietary counselling, type 2 diabetes mellitus, fasting blood glucose, HbA1c

## Abstract

**Background:**

The impact of dietary factors on glycaemic control in type 2 diabetes mellitus (T2DM) is well established. However, the effectiveness of transforming portion control into a practical innovation for glycaemic control in T2DM has not yet been established for counselling in nutrition. The aim of this study was to compare the effect of general counselling in nutrition (GCN) and a portioned meal box (PMB) on fasting blood glucose, glycated haemoglobin (HbA1c) and body composition.

**Methods:**

A randomised, parallel intervention trial was conducted over 12 weeks, with GCN: carbohydrate portion control concept by using food exchange lists (*n* = 25) and PMB: portioned meal box was set by energy requirements (*n* = 25).

**Results:**

Both GCN and PMB demonstrated reductions in HbA1c levels at the 6th and 12th weeks compared to baseline. However, no significant difference in HbA1c was observed between GCN and PMB at either the 6th or 12th week. Using PMB at least four times a week significantly decreased HbA1c during the intervention period (*p* = 0.021 and *p* < 0.001 for weeks 6 and 12 when compared with baseline, respectively). Changes in body composition were observed: body weight decrease in PMB only, body fat decrease and constant muscle mass in both groups. Both methods tended to relieve hunger and increased satiety in both groups. The satisfaction evaluation showed that participants preferred to use PMB over GCN (*p* = 0.001). Additionally, participants consumed less energy, carbohydrate and fat in PMB (*p* = 0.001, *p* = 0.019, and *p* = 0.001, respectively) and less energy and fat in GCN (*p* = 0.006 and *p* = 0.001, respectively).

**Conclusion:**

A better diet, either through GCN or PMB, can play an important role in improving dietary intake compliance and controlling blood glucose.

## 1. Introduction

Type 2 Diabetes Mellitus (T2DM) is the most common type of chronic disease for which genetic inheritances and environmental factors including lifestyle, dietary habits and physical activity are contributing factors (1, 2). Successful management of people with diabetes requires coordination of a multidisciplinary care team (MDT) (3), including endocrinologists, nurses, pharmacists and dietitians (4), to control glycaemia within a desirable range and, subsequently, to prevent diabetic complications of organs such as the eyes (5), kidneys (6) and feet (7). Blood glucose monitoring and body composition measurement are necessary to observe the responses to diabetic treatment and are used widely in research studies and the clinical setting (8). Strategies for diabetes intervention consists of four aspects, including optimal diet control, good mental health, exercise and compliance with medication usage (9, 10). Appropriate diet plays a vital role in maintaining good control of blood glucose concentration and keeping hunger and satiety levels in a desirable range in diabetes patients (11, 12). There are several approaches about dietary counselling for diabetes control in patients (13), for example, carbohydrate counting (14), low glycaemic index food (15) and portioned sizes control (16). Portion control is an effective approach for helping those with diabetes to avoid excessive macronutrient intake, especially carbohydrate content which is important to reduce blood glucose in diabetic patients (17). The plate model first proposed (in 1998) by the Swedish Diabetic Association uses pictures, graphs, and food replicas (18). The evidence indicates that portion control plates help promote healthy eating and nutrition knowledge and help to achieve weight loss (19–22). Although the MyPlate model fractions the plate as 30% of non-starchy vegetables, 20% of fruits, 25% of lean meat and 25% of whole grains (23, 24), most other portion-control plates follow a common proportion dedicating a quarter to protein-rich foods, a quarter to carbohydrates, and a half to vegetables (25). Due to the limited research available on portion plates and health promotion, we adapted the concept of portion-control plates to a portioned meal box (PMB), which is a practical, easy-to-use alternative in which the available space in the box for each food portion is evident. In Thai main meals and snacks, rice and rice products are commonly consumed as staple foods (26). However, diabetic patients often lack knowledge of the precise amount of carbohydrates in each meal (27), which hinders their ability to maintain desirable blood glucose levels (28). This can lead to poor glycaemic control, increased healthcare costs, uncontrolled diabetic complications, higher doses of oral hypoglycaemic medication (29) and increased mortality (30). Therefore, this study aimed to compare the effectiveness of the PMB portion-control concept with that of general dietary counselling (GCN) in improving blood glucose levels and body compositions. In addition, satisfaction, hunger and satiety levels, and dietary assessment parameters including food record were also investigated in diabetic patients.

## 2. Materials and methods

### 2.1. Study design and participants

The study was conducted at Burapha University Hospital in Chon Buri, Thailand, and the participants were diagnosed with type 2 diabetes (T2DM) at the out-patient department (OPD) on diabetes mellitus (DM). Throughout the study period, the participants maintained their antidiabetic medication regimen. The study enrolled participants who met the following inclusion criteria: aged between 30 and 60 years, on oral hypoglycaemic medication only (excluding insulin injections) and stable on the same medication for at least 1 month, and had an HbA1c level of more than 58.5 mmol/mol in the previous 6 months. Exclusion criteria included a change from oral hypoglycaemic medication to insulin injection, critical illness, steroid use, smoking or alcohol consumption, and participation in other studies that could affect blood glucose levels. The study was conducted from early November 2021 to mid-June 2022.

### 2.2. Sample size calculation

We calculated the required sample size for a parallel design trial comparing general dietary counselling and PMB in terms of HbA1c changes. A statistically significant difference was set at 95%, and the power of the test at 80%. We estimated the effect size of dietary counselling and portioned control diet to be a reduction of HbA1c by 0.02 ± 1.14% and 0.6 ± 0.80%, respectively, based on Pedersen’s (31) and Barnard’s studies (32). The equation (33) for sample size calculation is shown below, in which *Z*_α/2_ = 1.96 (*α* = 0.05), *Z*_β_ = 0.842 (*β* = 0.80), standard deviation (SD) = 0.97% and ∆Mean = (0.6–0.02%) = 0.58%.

$\begin{array}{l} n/\mathrm{group}={\left[\left(Z_{\pm /2}+Z_{2}\right)\;\mathrm{SD}/\Delta \mathrm{Mean}\right]}^{2}\\ \;\;\;\;\;\;\;\;\;\;\;\;\;\;\;\;\;\;\;\;\;\;\;={\left[\left(1.96+0.842\right)0.97/0.58\right]}^{2}\\ \;\;\;\;\;\;\;\;\;\;\;\;\;\;\;\;\;\;\;\;\;\;\;=23\;\mathrm{individuals}/\mathrm{group} \end{array}$

To allow for a potential dropout rate of participants, an additional 20% of participants were recruited resulting in 28 individuals per group.

### 2.3. Ethical considerations

All participants received study information and gave their written consent before beginning the study. This study was approved by Burapha University Ethical Committee with project ID HS014/2564 on 20 May 2021. All study protocols were performed according to the Declaration of Helsinki, and results were expressed by using the Consolidated Standards of Reporting Trials (CONSORT) guidelines (34). The methodology of the study was registered at the Thai Clinical Trials Registry (identification number: TCTR20221103006).

### 2.4. Study protocol

**Figure 1:**
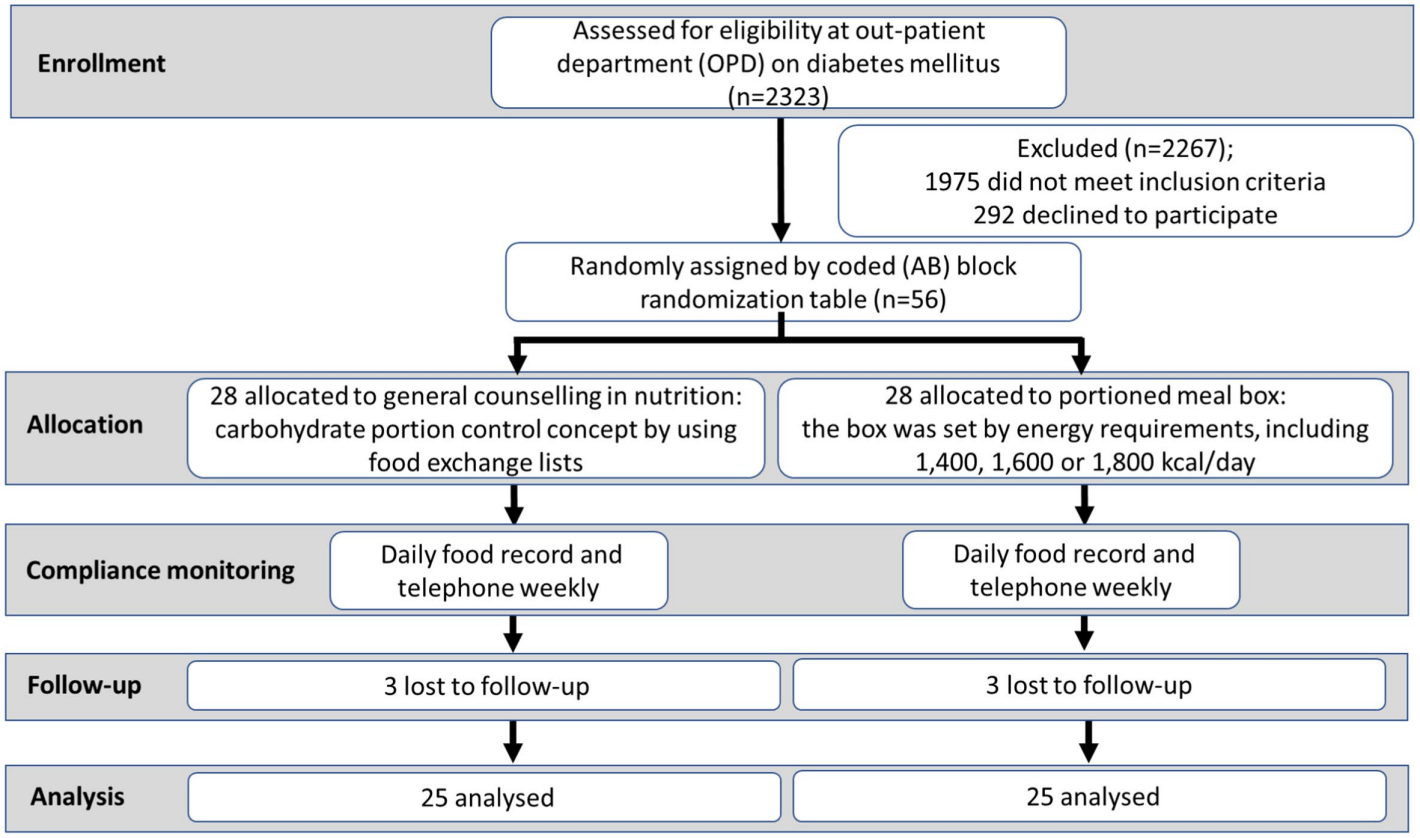
Study flowchart.

Participants were randomly allocated to either the intervention or control groups. Convenience sampling was used to recruit subjects for the study. Subject numbers for PMB and GCN were assigned based on a coded (AB) block randomisation table prepared by an independent statistician and group allocation was not disclosed until the first intervention day. The investigators were blinded to the randomisation table, code assignments, and procedure. Participants in the control group received personalised GCN that utilised the carbohydrate portion control concept by using food exchange lists. On the other hand, participants in the intervention group received two PMBs (Supplementary Figure 1) directly from the researcher. It is important to note that both groups received dietary guidance that was compatible with their calorie and carbohydrate requirements. All participants were instructed to generally exercise at least 30 min/day. Telephone calls were made weekly to all participants in each group to check their compliance and solve problems they may have encountered during the study period. An as-treated analysis was employed in this study to account for participant withdrawals during the course of the research. A flow diagram of a participant’s progress through the study is shown in Figure 1.

### 2.5. Portioned meal box

Portioned meal box (PMB) based on the healthy diet plate model concept to create an appropriate portion control, which relied on the participant’s requirements, including three parts (1/2 vegetables, 1/4 grains, and 1/4 meat). The calorie intake of each participant who used PMB was set by their personal requirements, including 1,400, 1,600 or 1,800 kcal/day.

### 2.6. Study parameters

At baseline, 6 weeks and 12 weeks, participants’ body compositions were measured namely, height (Ht), body weight (BW), body mass index (BMI), percent body fat (PBF), skeletal muscle mass (SMM), total body water (TBW), visceral fat level (VFL) by bioelectrical impedance analysis (Inbody 270, Biospace Corp., Seoul, South Korea), and waist to hip ratio (WHR) was calculated. Glycaemic control included fasting blood glucose (FBG) and HbA1c was measured in venous blood. According to manufacturer’s information, a rating of VFL between 1 and 12 indicates a health level of visceral fat, while a rating of VFL between 13 and 59 indicates excess visceral fat. Furthermore, hunger and satiety levels were evaluated using a visual analogue scale (VAS) (35, 36) immediately after the first meal and again 3 h later (Supplementary Figures 2, 3). All participants were instructed to maintain food diaries and record all foods and beverages consumed throughout the duration of the study. The nutrient composition, including energy, protein, carbohydrates, and fat, of each participant’s meal, was determined using INMUCAL Nutrients V. 2.0 (Mahidol University, Thailand), based on their daily food record (37).

### 2.7. Statistical analysis

All descriptive data were collected in Microsoft Office Excel 2019 and statistical analysis was performed by the IBM SPSS Statistics version 20.0. Results are represented as mean ± standard deviation (SD). Variations of monitored parameters between groups (GCN and PMB), including FBG, HbA1c, BW, BMI, PBF, SMM, WHR, VFL, and WHR at baseline, 6th week and 12th week were determined using one-way analysis of covariance (ANCOVA) with BMI and PBF subgroup analysis. Furthermore, the variations of HbA1c level and body composition for PMB used were analysed using ANCOVA with BMI and PBF as confounding factors. The difference between each treatment within groups and VAS for hunger and satiety was analysed using paired sample *t*-tests. Nutrient intake and satisfaction evaluation in participants were compared between groups by independent sample *t*-test.

## 3. Results

### 3.1. Baseline characteristics

Out of the initial total of 56 participants (28 in each group), three individuals dropped out from the intervention group (PMB) and three individuals dropped out from the control group (GCN) by the end of the 12-week study period due to reasons such as loss to follow-up and being uncontactable. At baseline, there were no significant differences between the two groups in terms of age, sex, BW, Ht, FBG, HbA1c, SMM, WHR, and TBW, as shown in Table 1.

**Table 1:** Participant baseline characteristics for control and intervention group.

Baseline parameters	Group		*p*-value
	Control (*n* = 25)	Intervention (*n* = 25)	
Age (years)	57.4 ± 8.3	54.8 ± 7.3	0.236
Sex [female (%)/male (%)]	16 (64)/9 (36)	18 (72)/7 (28)	0.554
BW (kg)	69.6 ± 8.3	77.9 ± 18.3	0.073
Ht (cm)	160.0 ± 8.6	160.0 ± 8.7	0.909
BMI (kg/m^2^)	26.9 ± 4.2	30.0 ± 6.2	0.050
FBG (mg/dL)	150.4 ± 52.5	145.1 ± 35.2	0.678
HbA1c (mmol/mol)	65.9 ± 8.4	67.7 ± 15	0.605
PBF (%)	34.1 ± 7.9	38.5 ± 7.6	0.050
SMM (kg)	24.9 ± 5.3	26.2 ± 5.6	0.417
WHR	0.9 ± 0.7	0.9 ± 0.1	0.280
VFL	11.3 ± 4.1	13.5 ± 5.2	0.095
TBW (kg)	33.2 ± 6.1	33.0 ± 6.4	0.901

Data are expressed as mean ± SD. BW, body weight; Ht, height; BMI, body mass index; FBG, fasting blood glucose; HbA1c, haemoglobin A1c; PBF, percent body fat; SMM, skeletal muscle mass; WHR, waist to hip ratio; VFL, visceral fat level; TBW, total body water.

### 3.2. Energy, carbohydrate, protein and fat intake in GCN and PMB

**Figure 2:**
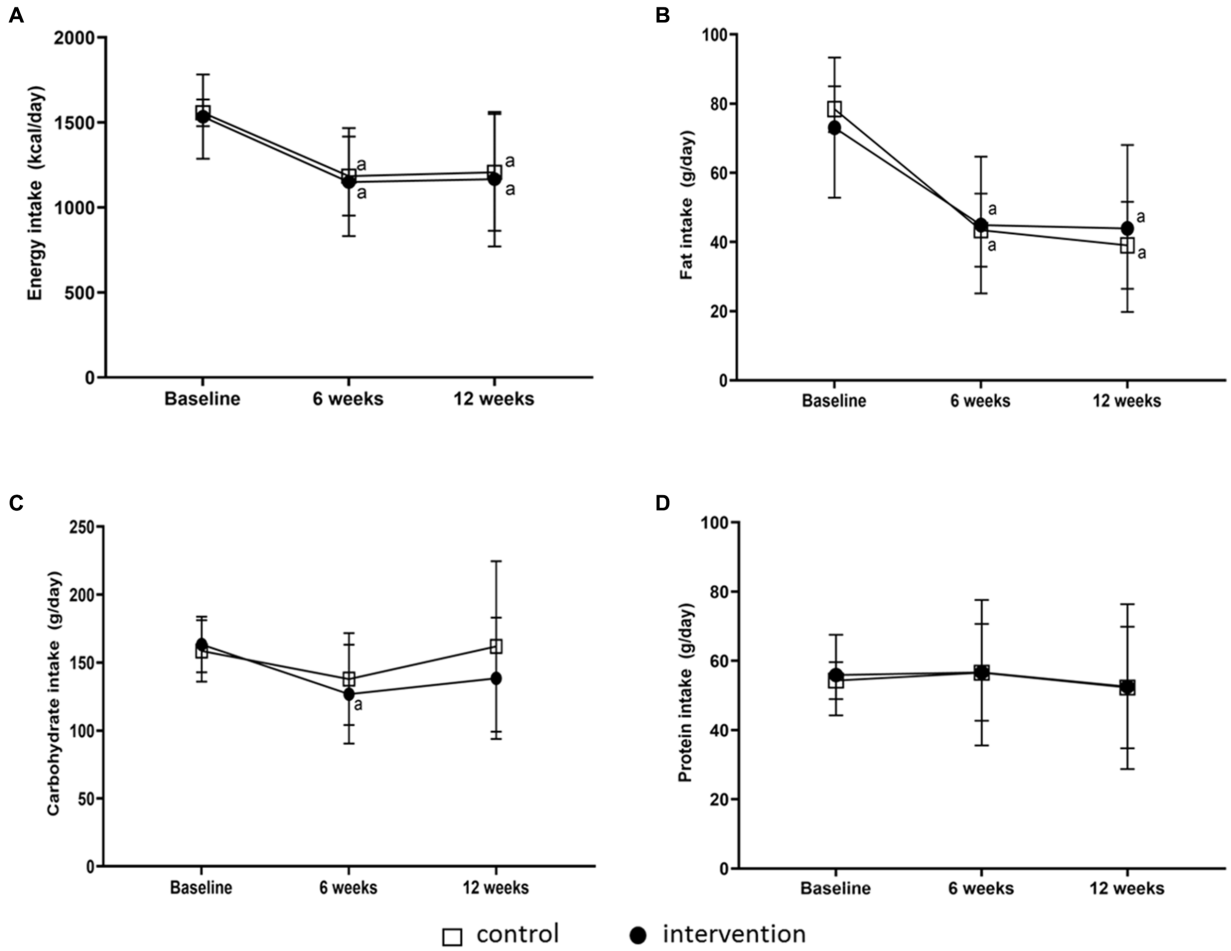
Changes in energy intake and nutrition intake compositions after 6 and 12 weeks of intervention. Energy **(A)**, fat **(B)**, carbohydrate **(C)** and protein **(D)** intakes in general dietary counselling (control) (*n* = 23) and portioned meal box (intervention) (*n* = 18) at baseline, 6^th^ week and 12^th^ week. ^a^ Different within the group when compared with baseline (*p* < 0.05).

At baseline, there were no significant differences in calorie intake between GCN and PMB (Supplementary Table 1). Both groups of participants (GCN and PMB) had reduced significantly their energy intake at weeks 6 (*p* < 0.001) and had lowered fat intake at weeks 6 (*p* < 0.001) and 12 (*p* = 0.001); (Figures 2A,B). Carbohydrate intake was decreased significantly in PMB at weeks 6 (*p* = 0.001) and 12 (*p* = 0.019); GCN: only weeks 6 (*p* = 0.020) (Figure 2C). However, there was no significant difference in protein intake within and between groups (Figure 2D and Supplementary Table 1). Both groups showed a significant decrease in daily energy intake after the 6th and 12th weeks, with the intervention group consuming less energy than the control group (105 and 153 kcal/day on weeks 6 and 12, respectively) (Figure 2A).

### 3.3. Effect of GCN and PMB on FBG and HbA1c

**Figure 3:**
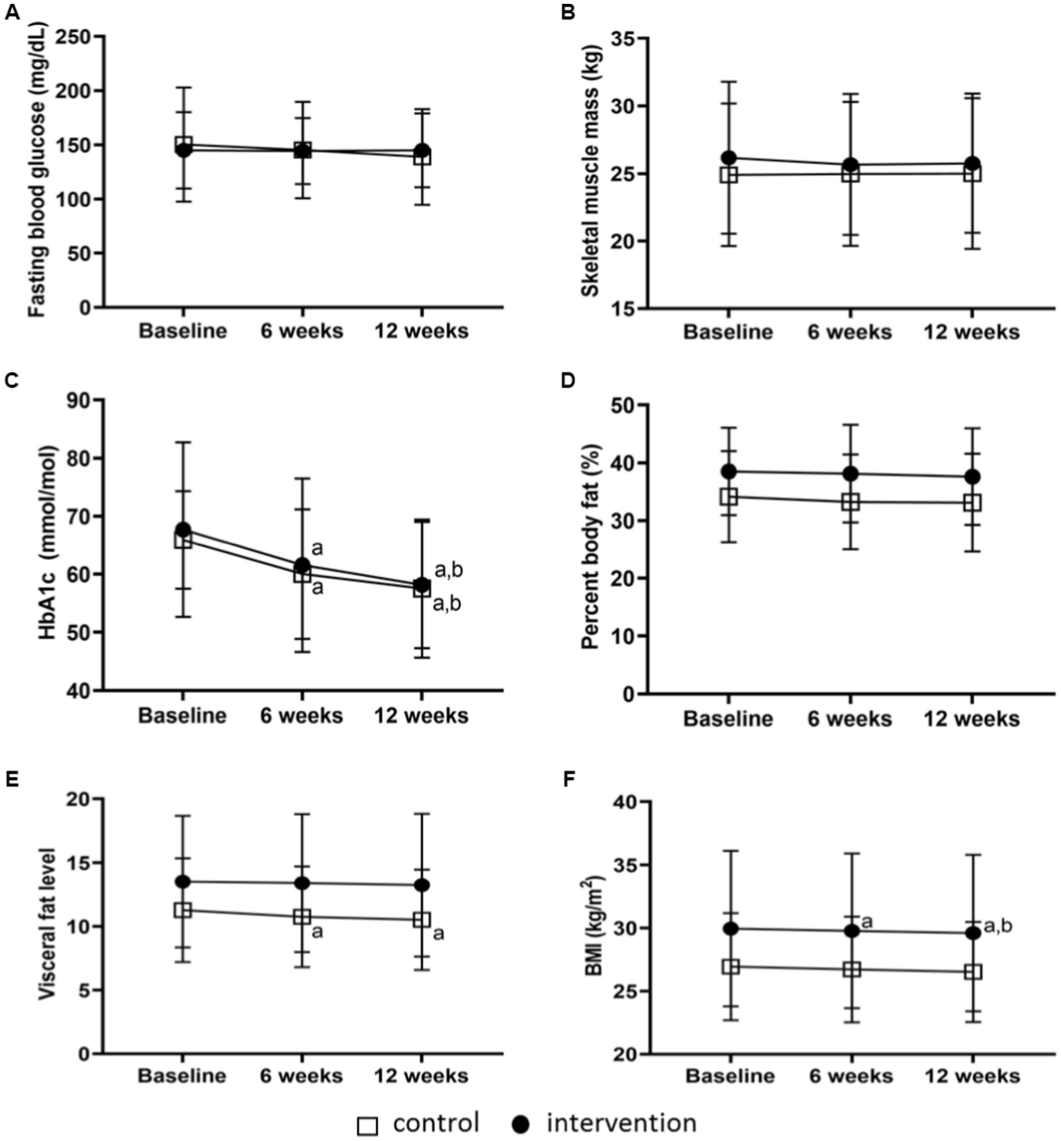
Glycaemic response and body composition trends at baseline, 6th week and 12th week. Fasting blood glucose **(A)**, skeletal muscle mass **(B)**, HbA1c **(C)**, percent body fat **(D)**, visceral fat level **(E)**, BMI **(F)** following general dietary counselling (control) (*n* = 23) and portioned meal box (intervention) (*n* = 18) at baseline, 6th week and 12th week. ^a^ Different from baseline for the same group (*p* < 0.05), ^b^ Different from week 6 for the same group (*p* < 0.05).

FBG showed variations neither between groups, nor over time (Table 2 and Figures 3A,B). After 6 and 12 weeks, all participants significantly decreased their HbA1c (Table 2 and Figures 3C–E). Interestingly, participants who received GCN and PMB showed significantly lower HbA1c levels at week 6 (60.0 ± 11.2 and 61.6 ± 14.9 mmol/mol) and week 12 (57.6 ± 11.9 and 58.2 ± 10.9 mmol/mol) when compared with baseline (*p* < 0.05), respectively. Although HbA1c levels did not vary between weeks 6 and 12 in PMB, PMB group showed a higher decrease in HbA1c levels (−9.5 ± 13.7 mmol/mol) compared with GCN (−8.4 ± 8.8 mmol/mol) (Table 2).

**Table 2:** Responses and changes of glycaemic and body composition for control group (*n* = 25) and intervention group (*n* = 25) at each time point when compared with baseline.

Parameters	6th week						12th week					
	Values		*p*-value	Differences (baseline-6th week)		*p*-value	Values		*p*-value			*p*-value
	Control	Intervention		Control	Intervention		Control	Intervention		Control	Intervention	
BW (kg)	69.2 ± 12.9	77.4 ± 18.2*	0.576	−0.6 ± 1.8	−0.5 ± 0.9	0.829	68.5 ± 12.5	76.9 ± 18.2*	0.549	−1.1 ± 3.4	−1.0 ± 1.8^†^	0.855
BMI (kg/m^2^)	26.7 ± 4.2	29.8 ± 6.1*	0.603	−0.2 ± 0.7	−0.2 ± 0.3	0.807	26.5 ± 4.0	29.6 ± 6.2*	0.545	−0.4 ± 1.2	−0.4 ± 0.7^†^	0.797
FBG (mg/dL)	145.0 ± 44.4	144.3 ± 30.3	0.754	−5.0 ± 29.3	−0.8 ± 30.7	0.611	139.0 ± 44.1	145.0 ± 34.1	0.438	−11.4 ± 47.8	−0.01 ± 39.5	0.376
HbA1c (mmol/mol)	60.0 ± 11.2*	61.6 ± 14.9*	0.987	−5.9 ± 7.4	−6.1 ± 8.3	0.898	58.2 ± 10.9*	57.6 ± 11.9*	0.848	−8.4 ± 8.8^†^	−9.5 ± 13.7	0.677
PBF (%)	33.2 ± 8.2	38.1 ± 8.5	0.79	−0.9 ± 2.3	−0.4 ± 2.8	0.483	33.1 ± 7.9	37.6 ± 8.4	0.855	−1.0 ± 2.7	−0.9 ± 2.3	0.849
SMM (kg)	25.0 ± 5.3	25.7 ± 5.2	0.239	+0.1 ± 0.9	−0.5 ± 1.8	0.146	25.0 ± 5.6	25.8 ± 5.2	0.183	0.0 ± 1.4	−0.4 ± 1.4	0.178
WHR	0.88 ± 0.06	0.91 ± 0.07	0.082	−0.02 ± 0.04	0.00 ± 0.01	0.11	0.87 ± 0.05*	0.90 ± 0.07	0.077	−0.03 ± 0.04^†^	−0.01 ± 0.02	0.092
VFL	10.8 ± 4.0*	13.4 ± 5.4	0.16	−0.5 ± 1.0	−0.1 ± 0.7	0.067	10.5 ± 4.0*	13.2 ± 5.6	0.332	−0.8 ± 1.5	−0.3 ± 1.0	0.168
TBW (kg)	35.4 ± 11.2	34.6 ± 6.2	0.406	−0.3 ± 1.7	−0.1 ± 0.7	0.694	35.4 ± 11.0	34.5 ± 6.2	0.411	−0.3 ± 1.9	−0.2 ± 1.3	0.701

All data are expressed as mean ± SD. Analysed by ANCOVA adjusted for PBF and BMI (comparison between group) and paired sample *t*-test (comparison within group). * Statistically significant difference of each parameter from baseline (*p* < 0.05), within the group. ^†^ Statistically significant difference from the baseline-6th week change (*p* < 0.05), within the group. BW, body weight; BMI, body mass index; FBG, fasting blood glucose; HbA1c, haemoglobin A1c; PBF, percent body fat; SMM, skeletal muscle mass; WHR, waist to hip ratio; VFL, visceral fat level; TBW, total body water.

### 3.4. Effect of GCN and PMB on body compositions

TBW, PBF and SMM showed no variations either between groups, or over time (Table 2 and Figures 3A,B). After 6 and 12 weeks, VFL had significantly decreased in GCN (*p* < 0.05) (Table 2 and Figures 3C–E). Only the PMB group had their BMI decreased in week 6 and 12 (Table 2 and Figure 3F). Variations of body compositions, including BW, BMI, PBF, WHR, SMM, VFL, TBW, and BMR, were not different between groups.

### 3.5. Relationship among the number of PMB usages, HbA1c levels and body compositions

The usage of PMB was variable within the intervention group, so we evaluated the number of times PMB was used to determine its relationship with HbA1c levels and body compositions. The usage rate of PMB in the intervention group showed that 12 participants used them moderately to regularly, 4–7 days per week, while 13 participants rarely used them, 0–3 days per week. The participants, regardless of their weekly use of PMB, reduced their FBG and HbA1c levels after 6 weeks by +13.4 ± 26.0, −16.1 ± 28.8 mg/dL and − 2.6 ± 4.1, −13.5 ± 10.4 mmol/mol and after 12 weeks by +14.6 ± 41.3, −15.9 ± 32.0 mg/dL and − 2.8 ± 6.0, −23.8 ± 15.0 mmol/mol (*p* < 0.05), respectively (Table 3). The more PMB usage, the stronger HbA1c decrease. Additionally, HbA1c levels dropped significantly in the intervention group when PMB was used 4–7 days/week (*p* < 0.001). Not only HbA1c was reduced but also other body composition values, including BW and BMI (Table 3).

**Table 3:** Variations of HbA1c level and body composition for PMB used.

Variations	PMB used 0–3 days/week (*n* = 13)	PMB used 4–7 days/week (*n* = 12)	*p*-value
	Difference (mean ± SD)	Difference (mean ± SD)	
FBG (mg/dL)			
Baseline-6 weeks	+13.4 ± 26.0	−16.1 ± 28.8	0.014*
Baseline-12 weeks	+14.6 ± 41.3	−15.9 ± 32.0	0.049*
6–12 weeks	+1.2 ± 37.4	+0.2 ± 9.2	0.922
HbA1c (mmol/mol)			
Baseline-6 weeks	−2.6 ± 4.1	−13.5 ± 10.4	0.021*
Baseline-12 weeks	−2.8 ± 6.0	−23.8 ± 15.0	0.000*
6–12 weeks	−0.2 ± 3.7	−10.3 ± 13.0	0.006*
BW (kg)			
Baseline-6 weeks	−0.3 ± 1.0	−0.7 ± 0.7	0.204
Baseline-12 weeks	−0.4 ± 2.0	−1.6 ± 1.4	0.115
6–12 weeks	−0.2 ± 1.2	−0.8 ± 0.8	0.111
BMI (kg/m^2^)			
Baseline-6 weeks	−0.1 ± 0.4	−0.3 ± 0.3	0.182
Baseline-12 weeks	−0.2 ± 0.7	−0.6 ± 0.5	0.107
6–12 weeks	−0.1 ± 0.5	−0.3 ± 0.3	0.115
PBF (%)			
Baseline-6 weeks	+0.3 ± 3.6	−1.2 ± 1.2	0.163
Baseline-12 weeks	−0.3 ± 2.6	−1.5 ± 1.9	0.207
6–12 weeks	−0.7 ± 3.0	−0.3 ± 1.5	0.671
WHR			
Baseline-6 weeks	−0.01 ± 0.02	0.00 ± 0.02	0.172
Baseline-12 weeks	0.00 ± 0.03	+0.01 ± 0.03	0.128
6–12 weeks	−0.01 ± 0.02	+0.01 ± 0.02	0.066
SMM (kg)			
Baseline-6 weeks	−1.07 ± 2.34	+0.12 ± 0.76	0.108
Baseline-12 weeks	−0.80 ± 1.75	+0.02 ± 0.70	0.146
6–12 weeks	+0.26 ± 1.59	−0.10 ± 0.73	0.462
VFL			
Baseline-6 weeks	−0.2 ± 0.4	0.0 ± 0.9	0.413
Baseline-12 weeks	−0.3 ± 1.0	−0.3 ± 1.1	0.892
6–12 weeks	−0.1 ± 0.6	−0.3 ± 0.5	0.441
TBW (kg)			
Baseline-6 weeks	−0.07 ± 0.77	−0.21 ± 0.69	0.628
Baseline-12 weeks	−0.09 ± 1.53	−0.23 ± 1.08	0.791
6–12 weeks	−0.02 ± 0.76	−0.02 ± 0.46	0.99

Analysed by independent sample *t*-test. * Statistically significant difference between groups (*p* < 0.05). A positive or negative value showed augmentation or reduction of the variable during each period. BW, body weight; BMI, body mass index; FBG, fasting blood glucose; HbA1c, haemoglobin A1c; PBF, percent body fat; SMM, skeletal muscle mass; WHR, waist to hip ratio; VFL, visceral fat level; TBW, total body water.

### 3.6. Evaluation of satisfaction, hunger and satiety levels after receiving diet from GCN and PMB

Participants in the PMB group differed significantly from those in the GCN group in overall pleasure, diabetic diet control perception and willingness to use PMB in the future (5.0 ± 0.2 v. 4.6 ± 0.5, *p* = 0.001), (4.9 ± 0.3 v. 4.4 ± 0.5, *p* < 0.001), (4.4 ± 0.5 v. 4.8 ± 0.4, *p* < 0.001) and (4.9 ± 0.3 v. 4.4 ± 0.5, *p* < 0.001), respectively (Supplementary Table 2). Moreover, there was a significant difference in the recommendation for both GCN and PMB to other people (4.8 v. 4.4, *p* = 0.009) (Supplementary Table 2). For hunger and satiety levels, there was a significant difference in fullness feeling (question 2, “How full do you feel?”) of the intervention group immediately after food intake and after 3 h (4.8 ± 1.3 v. 3.6 ± 1.3, *p* = 0.004) (Table 4). However, the analysis did not reveal any significant difference between GCN and PMB in relation to hunger and satiety levels for all the questions examined.

**Table 4:** Hunger and satiety levels in the first time of dietary control by GCN or portion meal box usage after diet intake immediately or 3 h later.

Hunger and satiety levels	Control (*n* = 25)		Intervention (*n* = 25)	
	Immediately	3 h after	Immediately	3 h after
1. How hungry do you feel?	3.3 ± 0.6	3.3 ± 0.6	3.4 ± 1.5	3.5 ± 1.2
2. How full do you feel?	4.3 ± 1.5	4.3 ± 1.5	4.8 ± 1.3	3.6 ± 1.3*
3. How invigorated do you feel?	5.0 ± 1.0	5.0 ± 1.0	4.9 ± 1.5	4.8 ± 1.6
4. How much do you think you could eat now?	3.7 ± 0.6	3.7 ± 0.6	3.4 ± 1.6	3.6 ± 0.9
5. How much do you feel an urge to eat?	3.3 ± 1.2	3.3 ± 1.2	3.3 ± 1.2	3.5 ± 1.1
6. How much are you preoccupied with thoughts of food?	2.3 ± 1.5	2.3 ± 1.5	2.5 ± 1.2	2.8 ± 1.2

Analysed by paired sample *t*-test. * Statistically significant difference within groups (*p* < 0.05). Each question was scored on a scale from 1 to 7, indicating low and high feeling, respectively.

## 4. Discussion

Eating too much or too little is subjective and often individuals have difficulty determining what an adequate portion is. In addition, individuals tend to consume more food when presented with larger packages (38). Thus, a portion-controlled diet has been used to control body weight and other body compositions in overweight and obese individuals. Portion control plates have been shown to be effective in supporting weight loss for individuals with obesity or overweight (21). Although both 2D model and 3D plate are effective for improving nutrition knowledge (19), they are not practical to measure precisely each portion of meat, grains, and vegetables. Therefore, in this study, we hypothesised that PMB (developed from the concept of portion control plates) would be effective in assisting patients with diabetes in improving portion control.

Our study revealed no significant differences in cardiometabolic parameters between the PMB and GCN. This lack of difference can be attributed to the similarity in nutrient intake composition between both groups and the relatively short duration of the intervention. However, our PMB allows individuals to tailor their meals easier by filling up the box according to the marked proportions. Additionally, it is practical for daily life and flexible for food choices, which may be more encouraging than a fixed diet (39). The results of this study showed that both GCN and PMB in T2DM patients reduced HbA1c on the 6th and 12th weeks despite using the same antidiabetic medications treatment at each visit. It is important to note that the reduction in HbA1c from baseline to the 12th week was 0.76% in the GCN group and 0.87% in the PMB group. Previous studies have shown that a reduction of 0.2% in HbA1c levels can lead to a 10% decrease in mortality (40, 41). Regarding the reduction in body weight, we observed a decrease of 1 kg. It is worth mentioning that the relationship between weight loss and HbA1c reduction may not follow a linear pattern, as demonstrated by Gummesson et al. in their study (42) where they found an estimated HbA1c mean reduction of 0.1% for every 1 kg of body weight reduction. Therefore, we hypothesise that the observed reductions in blood glucose concentration in these diabetic patients were due to: (1) the significantly diminished energy, carbohydrate and fat intakes by both groups between baseline and the 12th week (43–45) and/or (2) the increased contribution made by protein intake (up from 15 to 20% of energy). The latter may indeed help to preserve SMM and improve blood glucose responsiveness (46). Our findings are compatible with an earlier study by Grammatikopoulou et al. on how an appropriate medical nutrition therapy has an important role in treating diabetic patients both prediabetes, gestational diabetes and T2DM (47). A possible mechanism of lowering HbA1c through PMB use could be via optimal energy, carbohydrate, protein and fat intake (48). Macronutrient distribution in the control and intervention groups was rearranged from baseline to the end of the study by a lower percentage of carbohydrate and fat intake with constant protein consumption. The results of a diabetic diet after receiving GCN or PMB, which reflects on the percentage of macronutrients intake, complied with the standards of medical care in diabetes guidelines from the American Diabetes Association (ADA) 2022 (carbohydrate 45–65%, protein 15–20%, and fat 20–35% of total calories) (49, 50). In this study, energy intake was dramatically lowered from baseline to the end of the study in the control (−23%) and intervention (−24%) triggering a positive response to reduce insulin resistance in participants (51). Moreover, carbohydrate contents in the control group nearly remained the same during the study period (+2%), but in the intervention group, carbohydrate intake diminished in the 6th week (−15%). In line with Wheatley and Haimoto’s suggestions, reduction of carbohydrate intake is strongly recommended for better glycaemic outcomes in T2DM (52, 53). A low carbohydrate diet can decrease HbA1c and help in weight control in T2DM and pre-diabetes (54). Additionally, a smaller carbohydrate intake is associated with enhanced glycaemic control in diabetes patients (55). The Scientific Advisory Committee on Nutrition recommends a lower carbohydrate diet over a short-term period (up to 6 months) to improve glycaemic control (56). Our study results are in line with this recommendation, as both GCN and PMB groups demonstrated a reduction in carbohydrate and fat intakes, leading to observable health benefits. By reducing carbohydrate intakes in moderate amounts (45–65% carbohydrate of total energy) (57), based on energy requirement from the rule of thumb equation (25–30 kcal/kg ideal body weight per day) (58) and carbohydrate consumption, high blood glucose levels can be reliably reduced resulting in a reduction or suppression of medication. It is essential to maintain an appropriate amount of carbohydrate content to address poor dietary habits resulting from a lack of knowledge about the diabetic diet (59).

Furthermore, we observed that low HbA1c levels depended on the number of PMB usages. Participants who used PMB more than 4 days a week had a better regulated blood glucose than with the usual dietary control. Moreover, smaller meal box sizes can encourage awareness and become a generalised recommendation to control the amount of food intake at each meal (60). Portion restriction has advocated for energy intake and adjusted food quantity and body composition (61). In terms of satisfaction evaluation, the results show that participants under the PMB method had more pleasure to use and commit more to it than GCN. This could be due to the PMB (feasible and portable) support of proper behaviour for better glycaemic control, in accordance with an earlier study on future innovation on portion control accepted by diabetic patients (62). In addition, hunger and satiety levels after using PMB can be suitably maintained by restrictive food intake behaviour (63). According to the experiment of Angelopoulos et al., a healthy diet can optimise hunger and satiety levels, which can be used for dietary control in the long term (64). This study effectively demonstrated the positive impact of increasing the frequency of using a portion-control tool on reducing HbA1c levels in patients with diabetes. However, it is crucial to acknowledge and consider certain limitations that may influence the interpretation of these findings. One notable limitation is the relatively small number of participants in both the PMB and GCN groups. This limited sample size may have affected the statistical power and generalisability of the results. Additionally, it was observed that participants receiving PMB lacked the motivation to use it every day, primarily due to time constraints related to meal preparation. Because both PMB and GNC groups received nutritional counselling, it is likely that their dietary intake may have been affected in a similar way, resulting in no observable differences in the variations of their respective energy and food intakes. Consequently, no significant differences in outcomes were observed between the PMB and GCN groups. To address this issue and enhance motivation among participants, it could be beneficial to utilise social media platforms to facilitate the sharing and exchange of ideas for creating different PMB meals. This approach could provide participants with a supportive community and inspire creativity in utilising the portion-control tool effectively. Furthermore, it is important to acknowledge that the study faced challenges in controlling participants’ diets, especially when they were outside of their home, which could have influenced the effectiveness of the intervention. To address this limitation, the provision of portable and convenient meal options that participants can easily carry with them while on-the-go could be a viable solution. By offering these options, participants could maintain adherence to the portion-control tool even in situations where their dietary choices are less controllable. This approach aims to provide practical and accessible solutions for participants to sustain their dietary habits and promote the desired outcomes of the intervention. Therefore, future study will have to evaluate the effectiveness of utilising PMB, specifically in terms of improvement in glycaemic control. This would provide valuable insights into the potential benefits of this intervention in helping individuals manage their blood glucose levels. Conducting such studies would contribute to make of PMB a better intervention tool.

## 5. Conclusion

This study represents the first intervention trial to compare the effect of PMB and GCN on dietary control compliance and glycaemic control in diabetic patients. While no significant difference in HbA1c reduction was observed between the PMB and GCN groups, it is important to consider the study findings in the broader context and explore other relevant outcomes to comprehensively evaluate the potential of PMB as an adjunct to dietary counselling for glycaemic control. Furthermore, it is essential to consider secondary outcomes, participant adherence, acceptability, and potential long-term effects when evaluating the overall impact of the PMB intervention. Additional studies with larger sample sizes and longer follow-up periods may provide further insights into the potential benefits or specific subgroups that may derive greater benefit from PMB.

## Data availability statement

The raw data supporting the conclusions of this article will be made available by the authors, without undue reservation.

## Ethics statement

The studies involving human participants were reviewed and approved by the Burapha University Ethical Committee with project ID HS014/2564. The patients/participants provided their written informed consent to participate in this study.

## Author contributions

CN mentored CS-L and contributed her time to provide advice including the conception and design of this research. The intervention was set up, participants were recruited, and all parameters were evaluated by T-uM, PC, SS, and PP. Analysis was performed by T-uM, NY, LW, BP, and AD, who also interpreted the data and drafted the manuscript under the supervision of CS-L and CN. T-uM and CS-L had primary responsibility for the final content, which was read and approved by all authors.

## Funding

This research project was supported by the Faculty of Allied Health Science, Burapha University (grant no. AHS04/2564).

## Conflict of interest

The authors declare that the research was conducted in the absence of any commercial or financial relationships that could be construed as a potential conflict of interest.

The reviewer LN declared a shared parent affiliation with the authors NY, LW, CN to the handling editor at time of review.

## Publisher’s note

All claims expressed in this article are solely those of the authors and do not necessarily represent those of their affiliated organizations, or those of the publisher, the editors and the reviewers. Any product that may be evaluated in this article, or claim that may be made by its manufacturer, is not guaranteed or endorsed by the publisher.
