# Supplementary material for: Optimising blood glucose control with portioned meal box in type 2 diabetes mellitus patients: a randomised control trial
Source: Front Nutr. 2023 Jul 18;10:1216753. doi: 10.3389/fnut.2023.1216753 (PMC10390788; doi:10.3389/fnut.2023.1216753)
Supplement: Supplementary file 1 [file Data_Sheet_1.docx]

**Supplementary Table 1.** Difference of nutrient intake compositions between control group (*n*=25) and intervention group (*n*=25) at baseline and 12^th^ week

| **Nutrient intake** | **Mean difference ±SED (Mean_int_ – Mean_con_)** | |
| --- | --- | --- |
|  | **Baseline** | **12^th^ week** |
| Energy (Kcal/d) | -2.1±90.8 | -153.2±187.9 |
| Carbohydrate (g/d) | 9.5±9.9 | -25.5±29.0 |
| Protein (g/d) | 0.1±4.3 | 4.3±9.3 |
| Fat (g/d) | -4.5±5.6 | -4.1±12.0 |

SED = Standard Error Difference, int = intervention, con = control

**Supplementary Table 2.** Satisfaction evaluation of participants for control group (*n*=25) and intervention group (*n*=25)

**12 wk**

| **Satisfaction evaluation** | **Control** | **Intervention** | ***p-value*** |
| --- | --- | --- | --- |
| 1. How much are you satisfied with this method compared with a general method? | 4.6±0.5 | 5.0±0.2 | 0.001* |
| 2. Are you satisfied with this method? | 4.4±0.5 | 4.9±0.3 | <0.001* |
| 3. Would you recommend this method to others? | 4.4±0.5 | 4.8±0.4 | 0.009* |
| 4. Would you use this method again in the future? | 4.4±0.5 | 4.9±0.3 | <0.001* |

Analysed by independent sample *t*-test. *represents a significant difference between groups (*p*<0.05). Each question was scored on a scale from 1 to 5, indicating negative and positive opinions, respectively.

**Supplementary** **Figure 1.** Portion meal box’s characteristic.


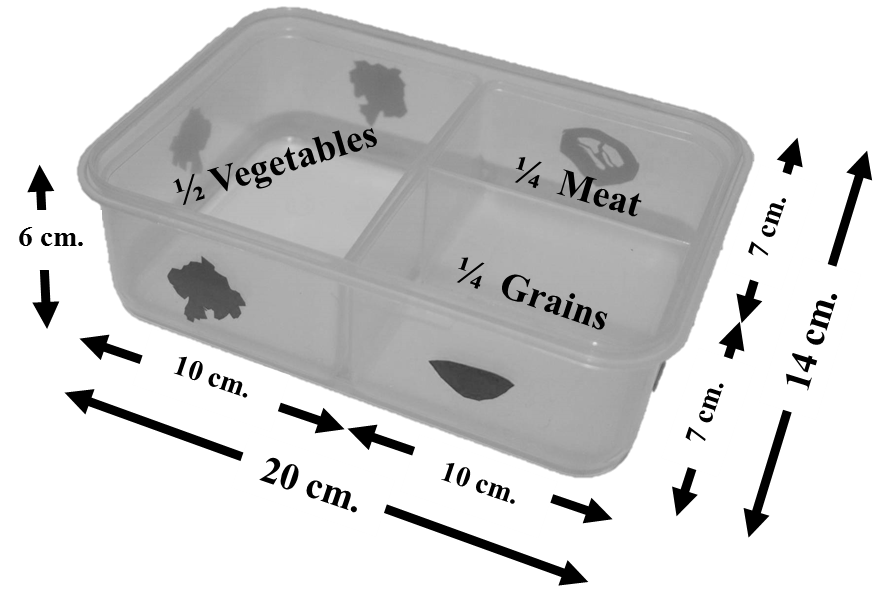


**Supplementary Figure 2.** Hunger and satiety level evaluation form (based after Hill & Blundell, 1982).

**Date:**

**Name: Sex (circle): Male/Female**

**Age: Body weight (kg): Height (cm):**

**Type and amount of foods: Evaluated after the first meal (circle):** immediately/ 3 hours later

| 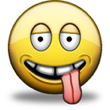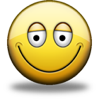**1. How hungry do you feel?**  1  2  3  4  5  6  7  (Not hungry at all Extremely hungry)  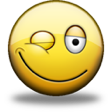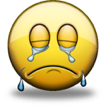**3. How invigorated do you feel?**  1  2  3  4  5  6  7  (Extremely fatigued Extremely energetic)  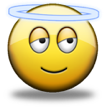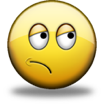**5. How much do you feel the urge to eat?**  1  2  3  4  5  6  7  (No urge Immediate need) | 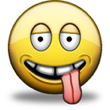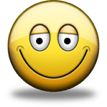**2. How full do you feel?**  1  2  3  4  5  6  7    (Completely empty Extremely full)  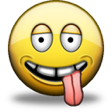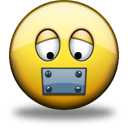**4.** **How much do you think you could eat now?**  1  2  3  4  5  6  7  (Nothing A lot)  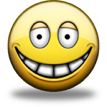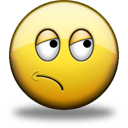**6. How much are you preoccupied with thoughts of food?**  1  2  3  4  5  6  7  (Not thinking Extremely anxious) |
| --- | --- |

**Supplementary Figure 3.** Satisfaction evaluation form on each diabetic diet education.

**1. How satisfied are you with this method compared with a general method (nutrition counselling)?**

🞎 5. Much more

🞎 4. More

🞎 3. About the same

🞎 2. Less

🞎 1. Much less

**2. Are you satisfied with this method?**

🞎 5. Very satisfied

🞎 4. Satisfied

🞎 3. Neither

🞎 2. Dissatisfied

🞎 1. Very dissatisfied

**3. Would you recommend this method to others?**

🞎 5. Definitely

🞎 4. Very likely

🞎 3. Possible

🞎 2. Probably not

🞎 1. Definitely not

**4. Would you use this method again in the future?**

🞎 5. Definitely

🞎 4. Very likely

🞎 3. Possible

🞎 2. Probably not

🞎 1. Definitely not
